# Genome-wide identification of brain miRNAs in response to high-intensity intermittent swimming training in *Rattus norvegicus* by deep sequencing

**DOI:** 10.1186/s12867-019-0120-4

**Published:** 2019-01-15

**Authors:** Yanhong Zhao, Anmin Zhang, Yanfang Wang, Shuping Hu, Ruiping Zhang, Shuaiwei Qian

**Affiliations:** 1 College of Agriculture, Ludong University, Yantai, China; 2 College of Sports, Yantai University, Yantai, China; 3 Institute of Health Sciences, Shanxi University of Finance & Economics, Taiyuan, China; 4 College of Life Sciences, Ludong University, Yantai, China

**Keywords:** Rat, miRNA, High-intensity intermittent swimming training, Exercise, Deep sequencing, Brain

## Abstract

**Background:**

Physical exercise can improve brain function by altering brain gene expression. The expression mechanisms underlying the brain’s response to exercise still remain unknown. miRNAs as vital regulators of gene expression may be involved in regulation of brain genes in response to exercise. However, as yet, very little is known about exercise-responsive miRNAs in brain.

**Results:**

We constructed two comparative small RNA libraries of rat brain from a high-intensity intermittent swimming training (HIST) group and a normal control (NC) group. Using deep sequencing and bioinformatics analysis, we identified 2109 (1700 from HIST, 1691 from NC) known and 55 (50 from HIST, 28 from NC) novel candidate miRNAs. Among them, 34 miRNAs were identified as significantly differentially expressed in response to HIST, 16 were up-regulated and 18 were down-regulated. The results showed that all members of mir-200 family were strongly up-regulated, implying mir-200 family may play very important roles in HIST response mechanisms of rat brain. A total of 955 potential target genes of these 34 exercise-responsive miRNAs were identified from rat genes. Most of them are directly involved in the development and regulatory function of brain or nerve. Many acknowledged exercise-responsive brain genes such as *Bdnf*, *Igf*-*1*, *Vgf*, *Ngf c*-*Fos,* and *Ntf3* etc. could be targeted by exercise-responsive miRNAs. Moreover, qRT-PCR and SABC immunohistochemical analysis further confirm the reliability of the expression of miRNAs and their targets.

**Conclusions:**

This study demonstrated that physical exercise could induce differential expression of rat brain miRNAs and 34 exercise-responsive miRNAs were identified in rat brain. Our results suggested that exercise-responsive miRNAs could play important roles in regulating gene expression of rat brain in response to exercise.

**Electronic supplementary material:**

The online version of this article (10.1186/s12867-019-0120-4) contains supplementary material, which is available to authorized users.

## Background

Accumulating evidence indicates that physical exercise helps maintain brain health and may profoundly benefit brain function [1–4], including the promotion of plasticity [2, 5], improvement of learning and memory performance [6, 7], mitigation of brain injury [8, 9] and reduction of brain disease risks (e.g., Alzheimer’s and Parkinson’s disease) [10, 11].

Previous studies have revealed that physical exercise elicits a differential gene expression pattern with significant changes in genes of relevance for brain function [12, 13]. For example, the expression level of brain-derived neurotrophic factor (BDNF) gene in various brain regions can be significantly increased after exercise [1, 14, 15], which is one of the major factors affecting on neuronal survival and differentiation as well as synaptic plasticity. Exercise can also rapidly increase gene expression of nerve growth factor (NGF) in specific areas of the brain [1]. Insulin-like growth factor 1 (IGF-1), a key regulator of reproductive neuroendocrine function, can be found at particularly high expression levels in brain after exercise [16, 17]. Moreover, many studies using animal models have demonstrated that physical exercise leads to differential expression of the brain genes encoding c-FOS [18, 19], NMDAR1 [20], VEGF [21, 22], Flk-1 [20], NT-3 [23], and VGF [24] etc. Such expression changes may contribute significantly to the beneficial effects of exercise [13]. However, it is still not clear how physical exercise alters gene expression profiling in brain.

The discovery of microRNA (miRNA) has greatly expanded our understanding of gene regulation mechanisms [25]. Many miRNAs have been identified from vertebrate brain and nervous system [26–28], and recent studies have demonstrated that miRNAs play critical roles in regulating gene expression during developmental processes like neurogenesis and neural differentiation and contribute to synaptic plasticity [29–33]. miRNAs also help protect against neurologic and psychiatric diseases [34, 35]. Interestingly, some miRNAs are also involved in regulating exercise-responsive brain genes, such as *Bdnf* [36], *Igf*-*1* [37], and *c*-*fos* [38]. These evidences provide new clues to insight into the mechanisms how physical exercise changes gene expression pattern in brain. This prompts us to hypothesize that physical exercise may induce differential expression of miRNAs in brain, so that exercise-responsive miRNAs further regulate gene expression in brain. However, as yet, very little is known about exercise-responsive miRNAs in brain.

The aim of this study was to determine genome-wide expression profiles of rat brain miRNAs in response to physical exercise by deep sequencing and verify whether physical exercise can induce differential expression of miRNAs in rat brain, and to further identify and analyze exercise-responsive miRNAs in rat brain. Our findings in this study may pave the way for further understanding the molecular mechanisms how exercise affects brain function from the perspective of miRNA regulation.

## Methods

### Experimental animals and exercise program

Male Wister rats (*R. norvegicus*) used in this study were provided by the Lab Animal Center of Shandong LVYE Pharmaceutical Co., Ltd (Yantai, China). All rats were housed in an environment control room (temperature 20–24 °C; relative humidity 40–60%; 12:12 h light/dark cycle) in groups of four or five rats per cage with food and water available ad libitum. Prior to beginning swimming training experiments, all rats were acclimated to swimming exercise once a day for 5 days (10 min, 15 min, 30 min, 45 min, and 60 min/day, respectively). Two-month-old healthy male rats (200–220 g) were then randomly assigned to a high-intensity intermittent swimming training (HIST) group (n = 62) and a normal control (NC) group (n = 62). All rats from the HIST group were conducted to high-intensity intermittent swimming training bearing a weight equivalent to 5% of their body weight for 6 weeks. Swimming exercise was performed in a swimming bath with 60-cm water depth and 35 °C water. In a 6-week training program, ten sessions of swimming training were performed each day for 6 min each with a 4-min rest period between each session. During the rest period, weights were removed and the rats were blow-dried and placed back to their cages. The rats of the NC group were kept sedentary in cages without swimming training.

After the 6-week experimental period, the rats were euthanized immediately by cervical dislocation under sodium pentobarbital anesthesia. The brain tissues (cerebrum and cerebellum together as a whole) of each rat were immediately dissected and collected. The samples were frozen in liquid nitrogen and stored at − 80 °C for later use.

### Small RNA library construction and deep sequencing

The brain tissues (comprised of cerebrum and cerebellum) of each rat were ground into power in liquid nitrogen. Total RNA was extracted using the TRIzol reagent (Invitrogen) following the manufacturer’s protocol. Fifteen RNA samples randomly selected from each group were mixed equally into HIST RNA pool and NC RNA pool, respectively. Small RNAs (sRNAs) of 16–30 nt were isolated and purified from the RNA pools by 15% denaturing PAGE. Subsequently, a 5′ RNA adaptor and a 3′ RNA adaptor were ligated to each sRNA using T4 RNA ligase. sRNAs were reversed transcribe into cDNA with SuperScript II Reverse Transcriptase (Invitrogen) and subjected to RT-PCR amplification. The RT-PCR products were further purified using 10% PAGE for constructing sRNA library. Lastly, HIST and NC sRNA libraries were sequenced in parallel by BGI (Shenzhen, China) using an Illumina/Solexa 1G Genome Analyzer (Illumina Inc., CA, USA).

### Analysis of the deep-sequencing dataset

Raw sequencing reads from deep sequencing were first processed to obtain high-quality clean reads with the length of 18–30 nt through the elimination of the following: low-quality reads, reads with 5′ primer contaminants, reads without 3′ primer, reads without the insertion tag, reads with polyA, and reads shorter than 18 nt. The clean reads were mapped to the rat genome using SOAP (developed by BGI) [39] to analyze their distribution on the genome developed by BGI [39]. To eliminate all other non-miRNA, the clean sequences were aligned against the Rfam and Genbank databases to and annotate into several classes of non-miRNA sequences (including mRNA, rRNA, tRNA, snRNA, snoRNA, srpRNA, scRNA, and repeat-associated RNA) based on priority by using tag2annotation program (developed by BGI). After these non-miRNA sequences were removed, the remaining clean reads were used for further miRNA identification.

### Identification of known miRNAs

The remaining sRNA sequences were searched against miRBase database (v18.0) to identify known miRNAs. The sRNA sequences matched to the known miRNAs of animal species deposited in miRBase database were considered to be known miRNAs in rat brain.

### Prediction of novel candidate miRNAs

To identify potential novel candidate miRNAs, the unannotated sRNAs tags were mapped back to the rat genome using SOAP program. Only perfect alignments were retained for predicting novel candidate miRNAs. The mapped sequences and their flanking sequences together were first extracted from the rat genome as candidate precursor sequences of novel miRNAs. The MIREAP program (developed by BGI) was employed to identify potential novel miRNAs by exploring the hairpin-like structure, the Dicer cleavage sites and the minimum free energy of the extracted candidate precursor sequences. Secondary structures of the candidate precursor sequences were also checked using Vienna RNA Package. Sequences that met the following criteria were then considered to be miRNA precursors: (1) the sequence can fold into an appropriate and stable stem-loop hairpin secondary structure with a lower minimal free energy (MFE ≤ − 25.0 kcal/mol) and a higher minimal free energy index (MFEI ≥ 0.85); (2) the mature miRNA is present on one arm of the hairpin precursor; (3) the mature miRNA strand and its complementary strand (miRNA*) form a duplex with 2-nt 3′ overhangs; (4) base-pairing between the miRNA and the other arm of the hairpin has no more than 4 mismatches; (5) there is no more than one internal loop or bulge within the miRNA/miRNA* duplex, and the bulge or loop in size is less than 2 bases; (6) the number of mature miRNAs with predicted hairpin must be no fewer than 5 in the alignment results.

### Differential expression analysis of miRNAs

To identify exercise-responsive miRNAs and to determine their genome-wide expression changes in response to HIST, we first compared the expression patterns of known and novel candidate miRNAs between HIST and NC libraries. miRNA counts in each libraries were first normalized to the number of transcripts per million (TPM). Then differential expression analysis of miRNAs between HIST and NC was performed using EXPR_SIG 3.0 tool (developed by BGI). Differential expression was considered to be significant when p-value < 0.05 and |log_2_(HIST/NC)| > 1. If the fold-change (log_2_(HIST/NC)) for a miRNA exceeded 1.0, it was considered to be up-regulated. If the fold-change was less than − 1.0, it was considered to be down-regulated miRNA.

### Prediction of target genes of the differentially expressed miRNAs

Bioinformatic approaches have been used in many studies as an effective strategy to predict miRNA targets [40]. Potential target genes of the differentially expressed miRNAs were predicted against a set of rat cDNA sequences using RNAhybrid [41], miRanda [42], and TargetScan [43]. When one target for a given miRNA was similarly predicted by at least two of the three prediction tools, it was considered as a putative target gene of the given miRNA. To annotate the potential target genes, we performed a BLAST comparison their sequences against the NCBI databases or directly extracted gene information from the *Rattus_norvegicus*.*gene_info* file downloaded from NCBI FTP site (ftp://ftp.ncbi.nih.gov/gene/DATA/GENE_INFO/Mammalia/Rattus_norvegicus.gene_info).

### qRT-PCR analysis

To further confirm the reliability of differential expressed analysis based on the sequencing results, twelve miRNAs (miR-4510, miR-182, miR-1839, miR-34c, miR-429, miR-122, miR-93, miR-212, miR-185, miR-7b, miR-483, and rno-miR-n048_5p) were selected to perform stem-loop qRT-PCR analysis.

Five rats were randomly sampled from each group for stem-loop qRT-PCR analysis. Each rat was an independent biological replicate. Small RNA (< 200 nt) was isolated from the brain region (consisted of cerebrum and cerebellum) of each rat using miRVana miRNA Isolation Kit (Ambion Inc., USA). First-strand cDNAs were synthesized with specific stem-loop RT primers (See Additional file 1: Table S1) using RevertAid First Stand cDNA Synthesis Kit (Thermo, Inc. USA) following the manufacturer’s protocol. Stem-loop qRT-PCR reactions were performed with miRNA-specific PCR primers (See Additional file 1: Table S1) and SYBR Green PCR mix (Qiagen) on a BioRad iCycler (BioRad, USA). U6 snRNA was used as an internal control and no-template qRT-PCR was used as negative control. Three technical replicates were done for each sample. The relative expression changes of miRNAs between HIST and NC were calculated using the 2^−ΔΔCT^ method [44]. Statistical comparison analysis of miRNAs relative expression levels was performed using SPSS. The difference of miRNA expression level between HIST and NC was considered as significant when *p *< 0.05.

In addition, we also selected five target genes (*c*-*Fos*, *Ncdn*, *Atrn*, *Ngf*, and *Ptn*) of exercise-responsive miRNAs to validate their expression patterns via qRT-PCR. Among them, *Ncdn*, *Atrn*, *Ngf*, and *Ptn* were targeted, respectively, by miR-141↑, miR-183↑, miR-3897-3p↓, and miR-2881↑. *c*-*Fos* was targeted by miR-483↓ and miR-7b↓. All primers are listed in Additional file 1: Table S1. Statistical comparison analysis was the same as the above method.

### SABC immunohistochemical analysis of c-Fos protein expression

Streptavidin-biotin complex (SABC) immunohistochemistry were applied to compare the expression of c-fos protein in rat brain tissues between HIST and NC. Rat brain tissues were cut into 5 μm sections using the freezing microtome. The sections were rinsed with 0.01 M PBS and incubated in turn with rabbit-anti-rat *c*-*Fos* polyclonal antibody (1:300; ZSGB-BIO company, China), biotinylated goat-anti-rabbit IgG (Boster Company, China), and streptavidin–biotin complex (SABC) (Boster Company, China) for 5 h, 30 min, and 30 min, respectively. The staining was visualized with diaminobenzidine (DAB) for 7–20 min at room temperature. The air-dried sections were dehydrated with graded ethanol and transparentized with xylene, and then sealed with neutral gum. Under the microscope (Olympus), the sections were observed and photographed for analyzing the density of *c*-*Fos* positive cells.

## Results

### Deep sequencing read analysis

**Fig. 1:**
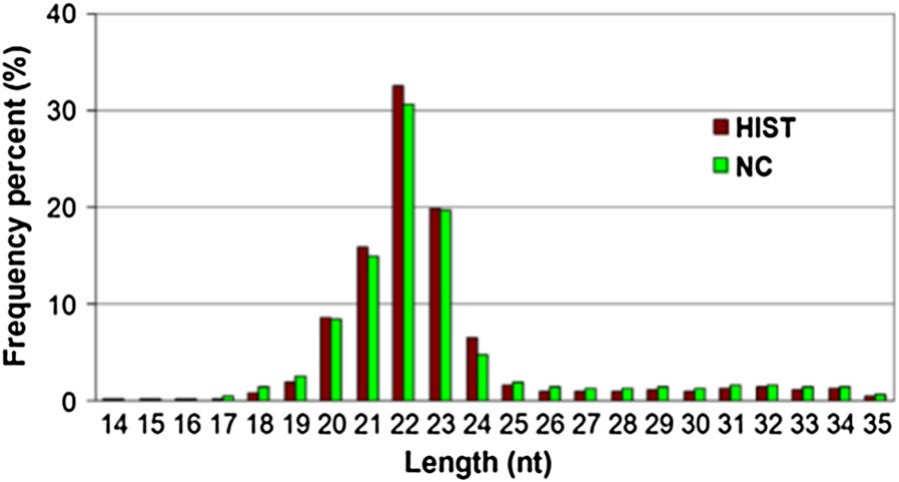
Length distribution of sRNA reads in HIST and NC libraries

**Fig. 2:**
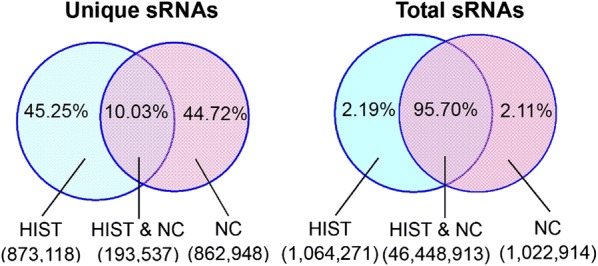
Summary of common and specific sRNA sequences between HIST and NC groups

Deep sequencing yielded a total of 28,667,031 HIST and 21,372,487 NC raw reads from HIST and NC libraries, respectively (Table 1). After filtering out low-quality reads, 3′ adaptor sequences were trimmed, contaminated reads were cleaned up, and reads shorter than 18 nt reads were removed, 27,877,781 HIST and 20,658,317 NC high-quality clean reads (≥ 18nt) were remained, corresponding to 1,066,655 HIST and 1,056,485 NC unique tags. The length distribution of sRNA sequences was similar in both libraries (Fig. 1). The majority (> 90%) of sRNA sequences were in the range of 18–25 nt. The 22-nt sequences were significantly more than those with other lengths, accounting for more than 30% of total clean reads. This length-distribution trend was consistent with the typical length-distribution trend of mature miRNAs in animals. Common sequences between HIST and NC libraries accounted for up to 95.7% of the total clean reads, representing 10.03% of all unique tags (Fig. 2). This indicated that common sRNAs between HIST and NC had higher expression levels than specific sRNAs. More than 70% of total clean sRNA reads from each library were mapped to the rat genome (Table 1), distributed across 21 chromosomes of rat.

**Table 1:** Summary statistics of small RNA deep sequencing

Library	Raw reads	High quality reads	Clean reads	Unique sRNAs	Total sRNAs mapped to rat genome	Unique sRNAs mapped to rat genome
HIST	28,667,031	28,122,819	27,877,781	1,066,655	20,847,175 (74.78%)	584,229 (54.77%)
NC	21,372,487	20,988,298	20,658,317	1,056,485	14,488,689 (70.13%)	594,634 (56.28%)

Among the clean sequences, a total of 4,789,460 HIST and 4,883,792 NC reads were annotated into several classes of non-miRNA sequences (i.e., mRNA, rRNA, tRNA, snRNA, snoRNA, srpRNA, scRNA, repeat-associated elements, etc.) (listed in Table 2). After these non-miRNA sequences were removed, the remaining 23,088,321 HIST and 15,774,525 NC clean reads were used for further miRNA analysis.

**Table 2:** Classification/annotation of small RNAs in HIST and NC libraries

Category	HIST sRNAs				NC sRNAs			
	Unique	(%)	Redundant	(%)	Unique	(%)	Redundant	(%)
rRNA	142,588	13.37	2,856,748	10.25	151,074	14.30	3,330,834	16.12
tRNA	42,591	3.99	1,143,993	4.10	34,900	3.30	789,023	3.82
scRNA	796	0.07	8178	0.03	777	0.07	6108	0.03
snRNA	6533	0.61	60,646	0.22	6648	0.63	59,169	0.29
snoRNA	7540	0.71	265,995	0.95	7049	0.67	236,933	1.15
srpRNA	1619	0.15	20,165	0.07	1633	0.15	22,995	0.11
exon_antisense	3517	0.33	4665	0.02	2999	0.28	3922	0.02
exon_sense	180,213	16.90	226,294	0.81	179,548	16.99	219,397	1.06
intron_antisense	11,691	1.10	14,701	0.05	10,414	0.99	12,811	0.06
intron_sense	81,952	7.68	121,328	0.44	93,129	8.81	128,385	0.62
Repeat	50,441	4.73	66,747	0.24	57,451	5.44	74,215	0.36
Unannotated	537,174	50.00	23,088,321	83.00	510,863	48.00	15,774,525	76.00
Total clean reads	1,0,66,655	100	27,877,781	100	1,056,485	100	20,658,317	100

### Identification of Known miRNAs in Rat Brain

**Fig. 3:**
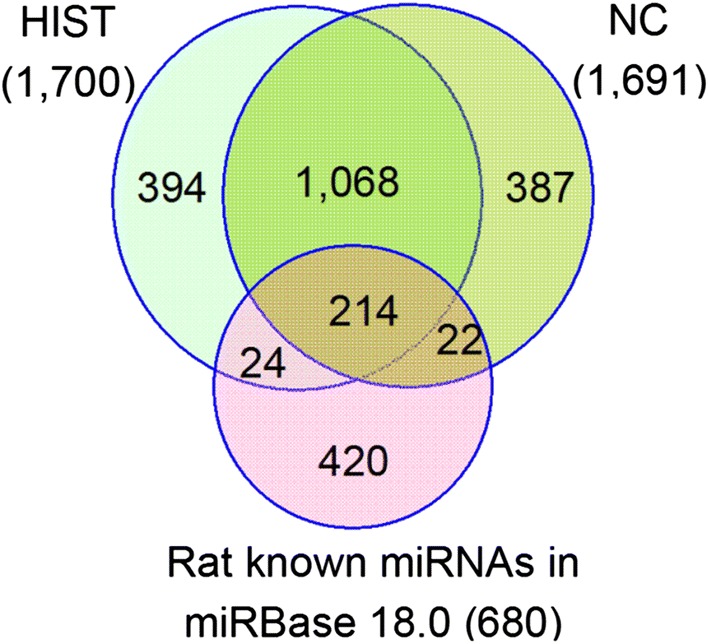
Co-expressed and specific-expressed known miRNAs between HIST and NC libraries and their matches to known rat miRNAs in miRBase 18.0

To identify known miRNAs in rat brain, the sRNA sequences from each library were aligned against all known miRNAs of *R. norvegicus* as well as other 90 animal species in miRBase database. We identified 2109 (1700 in HIST and 1691 in NC) known miRNAs, belonging to 434 miRNA families (Additional file 2: Table S2). 22,240,775 (79.8%) HIST and 14,954,364 (72.4%) NC clean reads were, respectively identified as known miRNAs, suggesting that majority of the isolated small RNAs were known miRNAs. The two libraries shared 1282miRNAs, which included 214 miRNAs matched to known miRNAs of *R. norvegicus* (Fig. 3). In our study, the identification of so many brain miRNAs indicated that miRNAs were likely to participate widely in gene regulation in rat brain. Among the 2109 known miRNAs, 260 miRNAs were matched to the known miRNAs of *R. norvegicus* in miRBase 18.0, which corresponded to 144 miRNA families and accounted for 38.2% of all known miRNAs (680 miRNAs) of *R. norvegicus*. The other miRNAs were matched to known miRNAs of 64 other animal species and about half of these miRNAs were matched to known miRNAs of *Mus musculus* or *Homo sapiens* indicating that the majority of miRNAs identified in this study were conserved across different animal species.

The known miRNAs in this study displayed significantly different expression levels as measured by the read counts, which ranged from one to more than one million copies (Additional file 2: Table S2). Twenty known miRNAs with the highest expression in the two libraries are listed in Table 3. Each of them is no less than 120,000 reads. However, 85 and 105 of 2109 miRNAs had only one copy in HIST and NC libraries, respectively. This indicated that Solexa sequencing technology is a powerful tool for identifying not only highly expressed miRNAs but also weakly expressed miRNAs. Among the identified 434 miRNA families, let-7 and mir-9 were two miRNA families with the highest expression in both libraries. In addition, thirty-five members of mir-154 family were identified in this study, more than those of other families.

**Table 3:** The most abundant known miRNAs obtained from HIST and NC libraries

miRNA name	Sequence	Length	Read count		miRNA family	miRNA orthologs
			HIST	NC		
let-7c	TGAGGTAGTAGGTTGTATGGTT	22	2,314,478	1,205,674	let-7	*hsa odi pma csa cin*
miR-9a-5p	TCTTTGGTTATCTAGCTGTATGA	23	1,889,962	1,416,714	mir-9	*dme*
let-7f	TGAGGTAGTAGATTGTATAGTT	22	1,754,130	1,414,639	let-7	*hsa*
miR-378	ACTGGACTTGGAGTCAGAAGGC	22	1,544,707	1,452,656	mir-378	*hsa ppy cfa*
miR-143	TGAGATGAAGCACTGTAGCT	20	1,526,043	1,139,450	mir-143	*rno mmu xtr pma*
miR-182	TTTGGCAATGGTAGAACTCACACCG	25	1,638,730	326,627	mir-182	*sko lgi cte mmu eca dre hsa spu*
miR-9	TCTTTGGTTATCTAGCTGTATG	22	1,103,879	751,193	mir-9	*egr sko bma xtr*
let-7	TGAGGTAGTAGGTTGTATAGTT	22	804,332	490,827	let-7	*cel aga sja spu*
miR-30a	TGTAAACATCCTCGACTGGAAGC	23	607,712	488,589	mir-30	*hsa dre oan*
miR-30d	TGTAAACATCCCCGACTGGAAGC	23	594,387	413,656	mir-30	*hsa pma oan bta*
let-7b	TGAGGTAGTAGGTTGTGTGGTT	22	638,961	307,685	let-7	*hsa odi pma xtr cin*
miR-127	TCGGATCCGTCTGAGCTTGGC	21	491,449	399,720	mir-127	*mmu oar*
miR-183	TATGGCACTGGTAGAATTCACT	22	623,293	125,748	mir-183	*lgi mmu gga sko csa*
let-7-5p	TGAGGTAGTAGGTTGTATAGT	21	339,133	220,610	let-7	*tca dme*
miR-103a	AGCAGCATTGTACAGGGCTATGA	23	280,569	274,894	mir-103	*hsa pma*
miR-24	TGGCTCAGTTCAGCAGGAACAGT	23	308,093	212,293	mir-24	*hsa cfa*
miR-26a	TTCAAGTAATCCAGGATAGG	20	285,016	218,341	mir-26	*hsa gga*
miR-379	TGGTAGACTATGGAACGTAGG	21	272,494	226,457	mir-379	*hsa*
miR-99b	CACCCGTAGAACCGACCTTGC	21	279,118	167,000	mir-99	*mmu*
let-7g	TGAGGTAGTAGTTTGTACAGTT	22	227,155	185,745	let-7	*mmu gga xtr dre*

### Discovery of novel candidate miRNAs in the rat brain

According to the described criteria and methods above, 55 putative novel miRNAs were identified from both libraries: 50 from HIST and 28 from NC (Table 4 and Additional file 3: Table S3). Among them, 23 were co-expressed in both libraries, 27 were identified only in HIST and 5 were found only in NC. We also found that the 55 novel miRNAs had a size range of 20–24 nt, which was similar to other known animal miRNAs. They were derived from 66 putative precursor sequences with hairpin structures, varying from 69 to 96 nt in length. The minimum free energy (MFE) of these hairpins varied from − 63.7 to − 25.8 kcal/mol with an average of − 40.9 kcal/mol. Their minimum free energy index (MFEI) ranged from 0.85 to 2.05 with an average of 1.14, which were significantly higher than that for mRNAs (0.62–0.66), rRNAs (0.59), and tRNAs (0.64).

**Table 4:** The most abundant novel candidate miRNAs identified from HIST and NC libraries

miRNA name	Sequence	Length	Count		Precursor location	MFE^a^	MFEI^b^
			HIST	NC			
rno-miR-n048_5p	CAGTGGTTTTACCCTATGGTAG	22	2605	2040	19:37422715:37422794:+	− 48.2	1.24
rno-miR-n026_5p	AATGTGACTCAGCTATCTGAAC	22	710	457	8:55466028:55466104:−	− 44.3	1.23
rno-miR-n031_3p	TCAGTAGGCCAGACAGCAAGC	21	588	453	11:36339323:36339403:−	− 39.6	0.86
rno-miR-n044_5p	TGTGTTTTGTGTGTGTACATGT	22	231	170	17:79266887:79266980:−	− 49.0	1.26
					17:79268511:79268591:−	− 33.8	1.06
					17:79270548:79270628:−	− 38.0	1.19
					17:79273909:79273989:−	− 38.0	1.15
					17:79275714:79275805:−	− 40.3	1.12
					17:79276506:79276586:+	− 38.0	1.15
					17:79281787:79281880:−	− 49.0	1.26
					17:79283453:79283546:−	-47.1	1.24
					17:79285357:79285437:−	− 33.8	1.06
					17:79277748:79277828:−	− 33.7	1.05
					17:79264421:79264501:−	− 38.0	1.19
rno-miR-n025_3p	ATGGTAATGGTGGTGGTGATGG	22	213	108	8:64686682:64686763:+	− 46.0	1.44
rno-miR-n002_3p	CATAAGTGTAGAGAGTCTGTAGT	23	122	59	1:131079839:131079925:+	− 31.3	0.95
rno-miR-n021_5p	TGGTTTACCGTCCCACATACA	21	77	57	6:134389818:134389888:+	− 43.5	1.45
rno-miR-n022_3p	AAGGGCAAGCTCTCTTCGAGG	21	46	36	6:134405230:134405322:+	− 42.7	1.07
rno-miR-n014_5p	TACAGTTAGACGTAGAGACCAT	22	49	31	3:153140628:153140705:−	− 35.0	1.21
rno-miR-n001_3p	CTCTAGCCAGGGCTTGACTGC	21	48	24	1:116347377:116347469:−	− 53.3	0.95
					1:116214989:116215081:+	− 54.6	0.99

^a^MFE denotes minimum free energy, and its unit is kcal/mol

^b^MFEI denotes minimum free energy index

These precursor sequences were distributed on all chromosomes except Chr13 (Additional file 3: Table S3). Chr1 and Chr17 contained more putative precursors than other chromosomes. The X chromosome had 4 of the 66 putative precursors. Among 55 novel candidate miRNAs, 53 had one unique genomic locus. However, rno-miR-n001-3p and rno-miR-n044_5p had multiple genomic loci. This indicated that different genomic loci might yield the same mature miRNA. Interestingly, rno-miR-n044_5p had 11 genomic loci, which distributed in cluster on Chr17. Further analysis revealed that all these 11 precursor sequences of rno-miR-n044_5p were almost identical. The existence of multi-copy miRNA genes suggests that the loss of function in one copy could be compensated by other copy or copies [45]. Because of the compensation mechanism, gene duplication can strengthen genetic robustness against null mutations to adapt the environment [45]. Meanwhile, this also implied that rno-miR-n044_5p might play pivotal regulatory roles in rat brain.

Compared with the known miRNAs, the predicted novel miRNAs exhibited much lower expression levels, ranging from 5 to 2605 copies (Additional file 3: Table S3). The majority of novel miRNAs (> 85%) were less than 100 reads. We found that 10 novel miRNAs with the highest expression were co-expressed in both libraries, together contributing more than 90% of the total expression of novel candidate miRNAs in each library (see Table 4). The reason that these novel miRNAs were not detected in previous studies could be that they express at the levels below the experimental detection threshold. Alternatively, they express only in some specific tissues at a particular developmental stage or under a specific induction.

### Differential expression of brain miRNAs in response to HIST

Deep sequencing technology enables genome-wide expression patterns of miRNAs at unprecedented quantitative and qualitative accuracy [46]. In this study, we constructed genome-wide expression patterns of miRNAs from HIST and NC based on high-throughput Solexa sequencing datasets (Additional file 2: Table S2 and Additional file 3: Table S3).

**Fig. 4:**
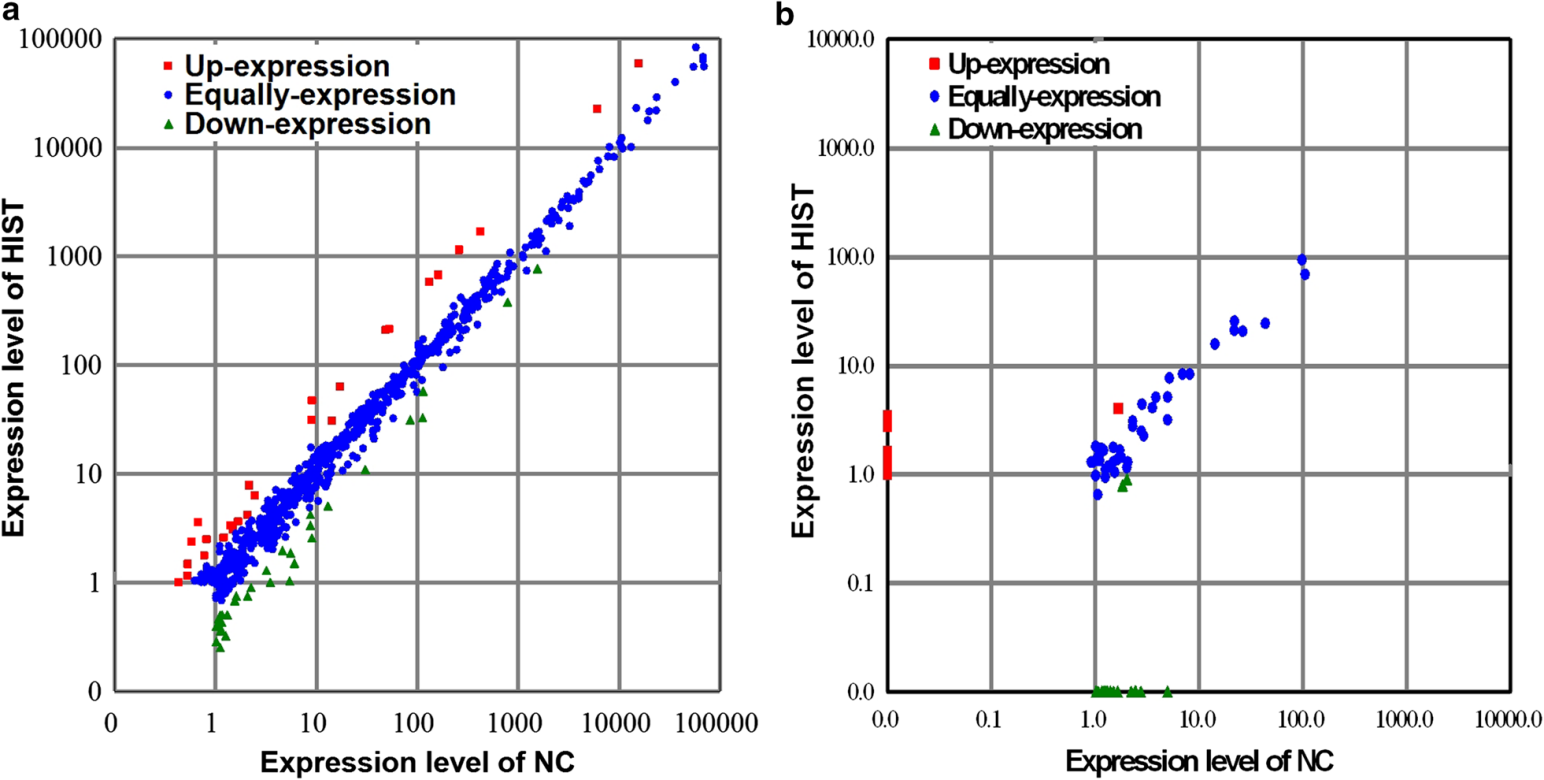
Scatter-plot graphs reveal the differential expression patterns of miRNAs between HIST and NC. **a** Scatter plot for known miRNAs. **b** Scatter plot for novel miRNAs

To investigate genome-wide expression changes of rat brain miRNAs in response to HIST and to screen exercise-responsive miRNAs in rat brain, differential expression analyses were performed to compare the expression patterns of known and novel miRNAs in rat brain between HIST and NC libraries based on the normalized reads. The majority of miRNAs showed similar expression patterns in both libraries. However, 31 known miRNAs and 3 novel miRNAs were identified as significantly differentially expressed between both libraries, with a more than two-fold change (|log_2_(HIST/NC)| > 1) and a *p*-value < 0.05. Among thirty-one differentially expressed known miRNAs, fifteen were up-regulated and sixteen were down-regulated (see Table 5, Fig. 4, and Additional file 4: Table S4). Of three differentially expressed novel miRNAs, rno-miR-n012_5p and rno-miR-n027_5p were down-regulated, and rno-miR-n006_3p was specifically expressed only in HIST library (see Table 5, Fig. 4, and Additional file 4: Table S4).

**Table 5:** Significantly differentially expressed miRNAs of the rat brain in response to HIST

miRNA name	HIST_Std^a^	NC_Std	log_2_(HIST/NC)	*p*-value	Sig.^b^	Mark^c^
miR-122	1.87	5.57	− 1.58	7.45E−03	**	Down
miR-1298	32.53	112.40	− 1.79	6.38E−18	**	Down
miR-1343	0.75	2.08	− 1.47	4.67E−02	*	Down
miR-141	214.33	52.96	2.02	1.01E−15	**	Up
miR-141*	3.55	0.68	2.39	3.24E−02	*	Up
miR-182	58,782.66	15,810.92	1.89	0	**	Up
miR-183	22,358.06	6087.04	1.88	0	**	Up
miR-200a	672.58	163.23	2.04	5.83E−46	**	Up
miR-200a*	31.14	9.00	1.79	1.83E−03	**	Up
miR-200b	1140.91	260.82	2.13	1.32E−81	**	Up
miR-200b*	62.85	17.28	1.86	1.44E−05	**	Up
miR-200c	1671.08	426.85	1.97	3.45E−103	**	Up
miR-263	7.75	2.18	1.83	3.27E−02	*	Up
miR-263a-5p	6.31	2.47	1.35	4.28E−02	*	Up
miR-2881	30.88	14.38	1.10	1.56E−02	*	Up
miR-34c	770.29	1543.64	− 1.00	8.67E−122	**	Down
miR-3897-3p	1.04	5.42	− 2.38	1.46E−02	*	Down
miR-4154-3p	3.34	8.76	− 1.39	6.13E−03	**	Down
miR-429	582.83	132.63	2.14	5.56E−43	**	Up
miR-4466	1.97	4.60	− 1.22	4.14E−02	*	Down
miR-448	31.42	84.91	− 1.43	6.27E−12	**	Down
miR-4492	1.00	3.49	− 1.79	4.92E−02	*	Down
miR-4497	10.98	30.54	− 1.48	1.29E−05	**	Down
miR-4508	2.58	9.05	− 1.81	2.33E−03	**	Down
miR-4510	46.78	9.10	2.36	6.53E−06	**	Up
miR-4651	1.51	6.05	− 2.01	7.08E−03	**	Down
miR-483	4.23	8.71	− 1.04	1.22E−02	*	Down
miR-5128	0.90	2.23	− 1.31	3.95E−02	*	Down
miR-7b	378.58	788.40	− 1.06	8.66E−67	**	Down
miR-84a	5.06	13.02	− 1.36	3.24E−03	**	Down
miR-96	208.98	48.99	2.09	2.47E−16	**	Up
rno-miR-n006_3p	1.51	0	7.24	8.58E−09	**	Up
rno-miR-n012_5p	0.79	1.84	− 1.22	1.89E−03	**	Down
rno-miR-n027_5p	0.90	2.03	− 1.18	3.94E−08	**	Down

^a^‘HIST_Std’ and ‘NC_Std’ denote the normalized expression of miRNAs in the HIST and NC libraries, respectively

^b^‘*’ and ‘**’ indicate significance at 0.05 and 0.01 probability level, respectively

^c^‘Down’ and ‘Up’ mean down-regulated and up-regulated miRNAs, respectively

Our results of differential expression analysis strongly confirmed that HIST can significantly change the expression patterns of miRNAs in rat brain. This suggested that these exercise-responsive miRNAs may play key roles in regulating gene expression of rat brain in response to HIST.

### miRNAs validation by stem-loop qRT-PCR

Twelve miRNAs with different expression levels were selected to perform stem-loop qRT-PCR analysis for validating their expression changes between HIST and NC. With the exception of miR-122, the other 11 miRNAs were successfully detected using stem-loop qRT-PCR. The lower expression level of miR-122 was the possible reason that it failed to be detected by stem-loop qRT-PCR.

**Fig. 5:**
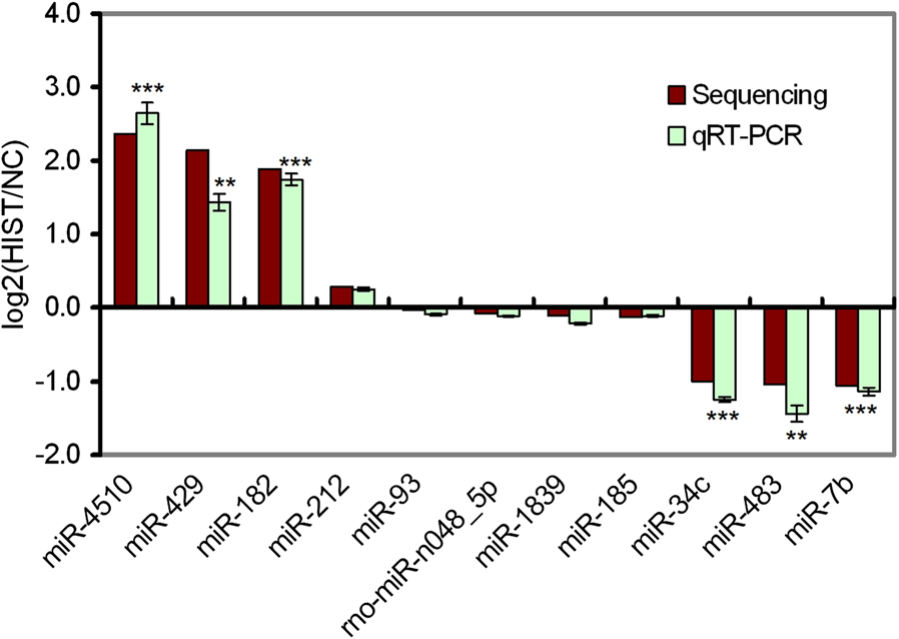
Validation of differentially expressed miRNAs by stem-loop qRT-PCR. The horizontal axis denotes the selected miRNAs for stem-loop qRT-PCR, and the vertical axis is the values of the Fold-change (log_2_(HIST/NC)) of miRNAs in response to HIST

The results of stem-loop qRT-PCR analysis showed that six miRNAs were significantly differentially expressed between HIST and NC, with a more than two-fold change (|log_2_(HIST/NC)| > 1) and a *p*-value < 0.05 (Fig. 5). Among them, three miRNAs (miR-4510, miR-429, and miR-182) were up-regulated and three miRNAs (miR-34c, miR-483, and miR-7b) were down-regulated. Five miRNAs (miR-212, miR-93, miR-1839, miR-185, and rno-miR-n048_5p) were not identified as significantly differentially expressed between both groups. The stem-loop qRT-PCR results were consistent with those of the differential expression analysis based on the deep sequencing data. Therefore, qRT-PCR analysis confirmed the reliability of the deep sequencing data and differential expression analysis.

### Target prediction of differentially expressed miRNAs

To further understand the potential regulatory functions of these significantly differentially expressed miRNAs in rat brain between HIST and NC, their target genes were computationally predicted by RNAhybrid, miRanda and TargetScan. A total of 946 potential target genes of 31 differentially expressed known miRNAs were identified from a set of transcript sequences corresponding to rat genes (see Table 6 and Additional file 5: Table S5). The number of targets per known miRNA varied from 1 to 149 with an average of approximately 38. Moreover, we also predicted targets of 3 differentially expressed novel candidate miRNAs and obtained 9 potential targets (Additional file 5: Table S5). The number of targets per novel miRNA varied from 1 to 6 with an average of approximately 3.

**Table 6:** The list of some target genes for exercise-responsive miRNAs in brain

Target genes	Gene ID	Description	miRNAs
*Igf1*	24482	Insulin-like growth factor 1	miR-483, miR-96
*Nefh*	24587	Neurofilament, heavy polypeptide	miR-4508
*Bdnf*	24225	Brain-derived neurotrophic factor	miR-183, miR-2881, miR-34c, miR-3897-3p, miR-483
*Ngfr*	24596	Nerve growth factor receptor	miR-141, miR-4492
*Rnf112*	24916	Brain ring finger protein 112	miR-182
*Ptn*	24924	Pleiotrophin	miR-2881
*Htr2c*	25187	5-Hydroxytryptamine (serotonin) receptor 2C	miR-4492
*Bcan*	25393	Brevican (brain specific proteoglycan)	miR-2881
*Htr5a*	25689	5-Hydroxytryptamine (serotonin) receptor 5A	miR-483
*Ntf4*	25730	Neurotrophin 4	miR-2881, miR-4492
*Neurod1*	29458	Neuronal differentiation 1	miR-4492
*Vgf*	29461	VGF nerve growth factor inducible	miR-2881, miR-3897-3p, miR-4492
*Htr2b*	29581	5-Hydroxytryptamine (serotonin) receptor 2B	miR-2881
*Cacnb1*	50688	Brain calcium channel, beta 1 subunit	miR-483
*Neurod2*	54276	Neuronal differentiation 2	miR-3897-3p, miR-4492
*Ntrk1*	59109	Neurotrophic tyrosine kinase, receptor, type 1	miR-3897-3p
*Basp1*	64160	Brain abundant, membrane attached signal protein 1	miR-2881, miR-3897-3p, miR-4492
*Smn1*	64301	Survival of motor neuron 1, telomeric	miR-4492
*Htr7*	65032	5-Hydroxytryptamine (serotonin) receptor 7	miR-2881
*Begain*	79146	Brain-enriched guanylate kinase-associated protein	miR-3897-3p
*Ntf3*	81737	Neurotrophin 3	miR-200b, miR-429
*Atrn*	83526	Membrane attracting	miR-183
*Ncdn*	89791	Neurochondrin(neurite outgrowth protein)	miR-141
*Slc17a7*	116638	Brain solute carrier family 17, member 7	miR-4154-3p
*Nfasc*	116690	Neurofascin	miR-182
*Baalc*	140720	Brain and acute leukemia, cytoplasmic	miR-34c
*Nlgn3*	171297	Neuroligin 3	miR-84a
*Nptx1*	266777	Neuronal pentraxin I	miR-182, miR-200b, miR-429
*Smndc1*	287768	Survival motor neuron domain containing 1	miR-2881
*Nptx2*	288475	Neuronal pentraxin II	miR-4492
*Npdc1*	296562	Neural proliferation, differentiation and control, 1	miR-4492
*Net1*	307098	Neuroepithelial cell transforming 1	miR-4492
*Ngf*	310738	Nerve growth factor (beta polypeptide)	miR-3897-3p
*c*-*Fos*	314322	c-fos oncogene	miR-483, miR-7b
*Ppp1r1b*	360616	Protein phosphatase 1, regulatory (inhibitor) subunit 1B	miR-1343, miR-2881, miR-4497, miR-4651
*Zc3h15*	362154	Zinc finger CCCH-type containing 15 in brain	miR-200c, miR-429

The results of target prediction indicated that these exercise-responsive miRNAs may participate in regulating many genes in rat brain. All of the predicted target genes encode proteins, and approximately one-third of them are transcription factors. Most of them are directly involved in the development and regulation of brain or nerve such as neurotrophin/neuropeptide, neural structure, neural signaling, synaptic protein, immune response, protein processing, metabolic enzyme, aging, and so on.

Neurotrophins are a class of essential proteins that induce the survival, neurogenesis, differentiation, development, and function of neurons. The neurotrophin family includes NGF, BDNF, NT-3, and NT-4 etc. NGF is critical for neuronal survival, maintenance and differentiation. Without it, neurons undergo apoptosis. NGF is a protein complex formed by three subunits (α, β, and γ). β is the biologically active subunit encoded by *Ngf* gene. *Ngf* gene was predicted to be targeted by miR-3897-3p↑. Moreover, *Ngfr*, a NGF receptor gene, was also targeted by miR-141↑ and miR-4492↓. *Bdnf* gene, encoding BDNF, was targeted by five exercise-responsive miRNAs (miR-183↑, miR-2881↑, miR-34c↓, miR-3897-3p↓, and miR-483↓). *Ntf3* and *Ntf4* encode the growth factors NT-3 and NT-4, respectively. They are expressed in certain neurons of the peripheral and central nervous system, and they help to support the survival and differentiation of existing neurons and promote the growth and differentiation of new neurons and synapses. *Ntf3* was a candidate target gene of miR-200b↑ and miR-429↑, and *Ntf4* was a candidate target of miR-2881↑ and miR-4492↓. This indicated that these exercise-responsive miRNAs participate in regulating the expression of neurotrophin genes in rat brain.

In addition, *Vgf*, a neuropeptide gene, was a shared candidate target gene of miR-2881↑, miR-4492↓, and miR-3897-3p↓. It may play a role in regulating energy homeostasis, metabolism [47] and synaptic plasticity [48] in rat brain. *Ncdn* (neurochondrin), a candidate gene targeted by miR-141↑, regulates neuronal synaptic plasticity. As an indirect marker of neuronal activity, *c*-*Fos* gene is the candidate target for miR-483↓ and miR-7b↓. Dopamine (DP) and 5-hydroxytryptamine (5-HT) are two important neurotransmitters. *Ppp1r1b(DARPP32),* a dopamine- and cyclic adenosine monophosphate-regulated phosphoprotein gene in rat brain, was a candidate target for four exercise-responsive miRNAs. 5-hydroxytryptamine receptor genes (*Htr2b*, *Htr2c*, *Htr5a*, and *Htr7*) were also candidate targets of exercise-responsive miRNAs. Among these predicted targets, *Nptx1* and *Nptx2* are involved in forming neural structure. This showed that these exercise-responsive miRNAs may play wide and important roles in the regulatory network of rat brain gene expression induced by HIST.

**Fig. 6:**
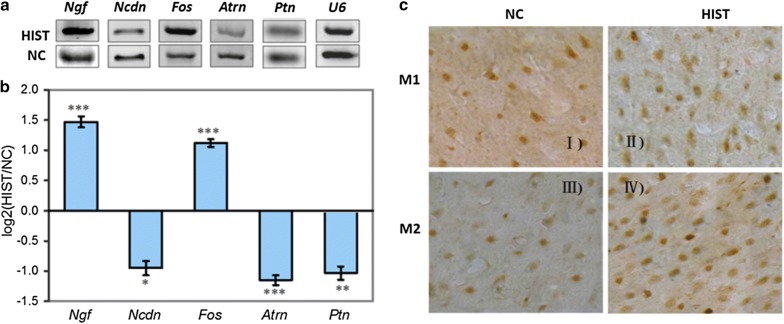
Validation of expression changes of miRNA targets in rat brain between HIST and NC by qRT-PCR and SABC immunohistochemistry. **a** Gel electrophoresis results of qRT-PCR for five target genes (*Ngf*, *Ncdn*, *Fos*, *Atrn*, and *Ptn*). Here, U6 snRNA was used as an internal control. **b** The expression fold changes of five target genes between HIST and NC detected by qRT-PCR. The vertical axis is the values of the Fold-change (log_2_(HIST/NC)) of target gene expression in response to HIST. **c** The immunohistochemistry results of brain c-fos protein expression changes between HIST and NC. Under the light microscope, c-Fos protein expression was detected in primary motor cortex (M1) and secondary motor cortex (M2) by the SABC immunohistochemistry techniques. II and IV are the c-Fos expression results in M1 and M2 regions of the HIST group, respectively. I and III are their corresponding controls. The number of Fos positive cells in M1 and M2 is significantly increased in response to HIST

The qRT-PCR results of five candidate target genes showed that three genes (*Ncdn*, *Atrn*, and *Ptn*) were down-regulated and two genes (*c*-*Fos* and *Ngf*) were up-regulated in response to HIST (Fig. 6a, b). Expression analysis revealed that the expression patterns of all selected candidate target genes were negatively correlated with their corresponding miRNAs, verifying the accuracy of target prediction.

SABC immunohistochemical analysis in rat brain confirmed that positive expression of *c*-*Fos* protein in HIST group was significantly higher than that in NC group (p < 0.01) (Fig. 6c). The number of *c*-*Fos* positive cells in HIST increased by a 2.1-fold in comparison to NC. By this token, the down-regulated expression of miR-483 and miR-7b, led to the expression increase of their target gene *c*-*Fos* at both mRNA and protein levels.

## Discussion

As a behavioral stimulus, physical exercise can affect brain health and plasticity by altering gene expression in brain [2, 13]. Insight into the regulatory mechanisms of brain genes in response to exercise stimulation can help us understand how exercise acts on the regulatory networks controlling brain function. In the past few years, miRNAs have turned out to be new players in regulating gene expression of animals or plants response to stimuli or stresses [49]. Previous reports have revealed that exercise stimulation has effects on miRNA expression in skeletal muscle [50, 51] and ventricular function [52] etc. Here, we found that exercise can change miRNA expression profile in rat brain.

In this study, genome-wide identification of rat brain miRNAs (including 2109 known miRNAs and 55 novel miRNAs) greatly enriched the population of brain miRNAs in rat. This also implied that the number of miRNAs in rat brain is likely to be larger than previously thought. Many brain miRNAs reported in previous studies were also detected in our study. We found that these previously reported brain miRNAs were generally highly expressed in our study. For instance, miR-9, let-7, and miR-127 had expression levels greater than 10,000 TPM in both libraries. Most of the 2109 miRNAs identified in our study were not brain-specific miRNAs. In previous reports, they were also identified from other tissues across different animal species. For example, miR-378 identified in this study was also strongly expressed in the mammalian heart [53].

Differential expression analysis demonstrated that HIST significantly changed the expression patterns of miRNAs in rat brain. Thirty-four differentially expressed miRNAs were identified as exercise-responsive miRNAs, involving in regulating the expression of brain genes in response to exercise. We found that most of the previously reported brain miRNAs with high expression level (> 1000 TPM) were not generally identified as significantly differentially expressed in response to HIST. A reason for this difference may be that we used the strict threshold (|log_2_(HIST/NC)| > 1 and *p*-value < 0.05). Many miRNAs with 1.5- to 2.0-fold change and a *p*-value < 0.05 were not considered to be significantly differentially expressed in our study. Of course, we also found that a few of brain-enriched miRNAs (miR-182, miR-183, miR-200c, and miR-7b etc.), previously reported to be associated with brain function, were identified as differential expressed miRNAs in response to exercise. In addition, a few of specific-expressed miRNAs in HIST or NC were not identified as exercise-responsive miRNAs. This was because their expression levels were too low (TPM < 1) to meet the needs for differential expression analysis.

It was worth noting that all members of mir-200 family (miR-200a, miR-200b, miR-200c, miR-141, and miR-429), together with their asterisk miRNAs (miR-200a*, miR-200b*, and miR-141*) were identified as significantly differentially expressed with more than 3.5-fold change, accounting for 23.5% (8/34) of all differentially expressed miRNAs. Their expression levels were strongly up-regulated in response to HIST. This implied that mir-200 family plays regulatory roles in rat brain in response to HIST. In previous studies, they have also been identified in the different tissues of some animal species [54–57]. Choi et al. found that mir-200 family could regulate olfactory neurogenesis [58]. Lee et al. reported that miR-200b and miR-200c had the strong neuroprotective effect [59]. Here, we also found mir-200 family members were involved in the development and differentiation of the brain and nerve (see Additional file 5: Table S5). Therefore, we believed that further exploration of the roles of mir-200 family in brain may be very important to understand the complex regulatory mechanism of gene expression responsive to exercise in rat brain.

Most of the predicted target genes for the exercise-responsive miRNAs were directly related to the brain or nerve function in rat, implying our target prediction was relatively reliable. Target prediction revealed that a miRNA often had multiple target genes while a target was often targeted by multiple miRNAs. For example, *Bdnf* gene was targeted by two up-regulated and three down-regulated miRNAs. Hence, in most cases the relationship between a miRNA and its target gene(s) may not be simple one-to-one, implying that the rat brain is subject to a complex and flexible network of miRNA-mediated gene regulation. However, this also made it difficult to experimentally validate miRNA-target interactions. In this study, we only selected some simple miRNA-target pairs to verify the accuracy of target prediction by qRT-PCR.

**Fig. 7:**
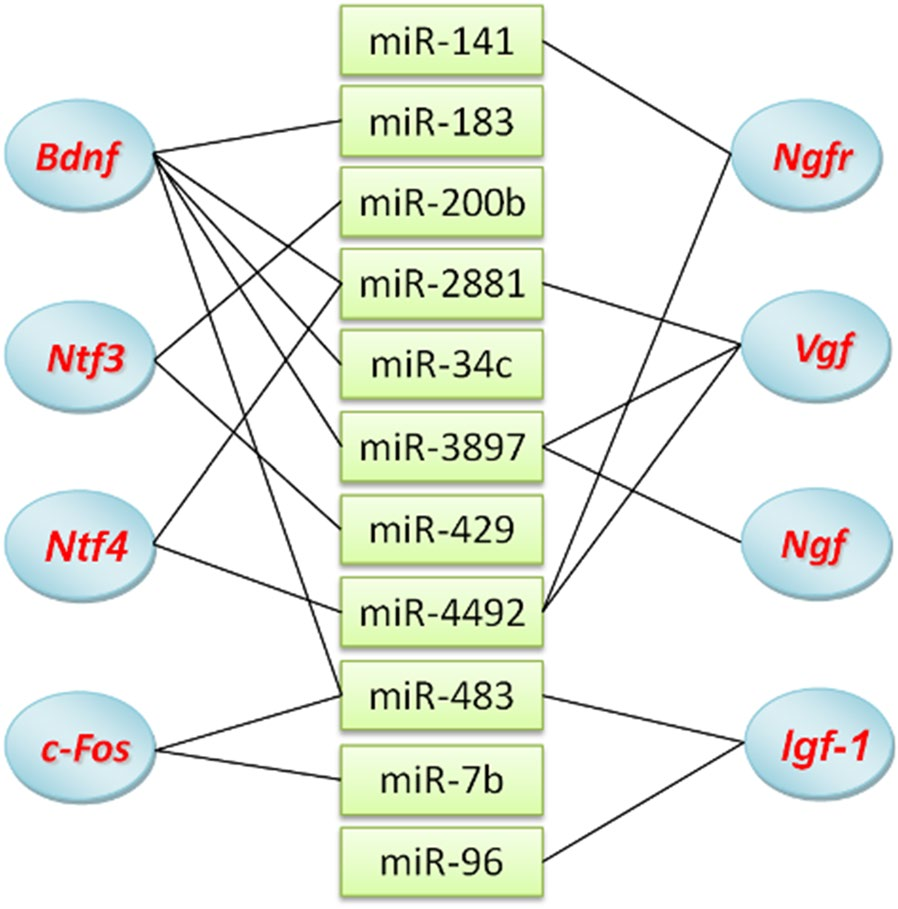
The corresponding relationship of the acknowledged exercise-responsive genes and the differentially expressed miRNAs

Interestingly, many exercise-responsive genes, including *Bdnf*,*Igf*-*1*, *Ngf*, *c*-*Fos*, *Ntf3*, *Ntf4*, and *Vgf* etc., could also be targeted by exercise-responsive miRNAs (See Fig. 7). *Igf*-*1* was targeted by miR-96 and miR-483, and *c*-*Fos* by miR-7b and miR-483. A previous study confirmed that up-regulated miR-7b inhibited the translation of c-*Fos* mRNA [38]. We also found that miR-483, a down-regulated miRNA, could simultaneously target three exercise-responsive genes: *Bdnf*, *Igf*-*1*, and *c*-*Fos*. These evidences revealed that the exercise-responsive miRNAs can participate in regulating the expression of the exercise-responsive genes in rat brain. Hereto, our results provided enough evidences to confirm that physical exercise can trigger differential expression of miRNAs in brain, so that exercise-responsive miRNAs can further stimulate changes in gene expression profile in brain.

Exercise programs with different intensities can induce different gene expression profiles in brain [20]. Hence, we postulated that exercise programs with differing intensities might also lead to intensity-specific changes in miRNA expression patterns. In addition, many previous reports have shown that the gene expression induced by physical exercise varies in different brain regions. Therefore, we postulated that miRNA expression patterns in response to exercise might also be different in different brain regions. This study only explored the effect of HIST on miRNA expression profiles in specific brain region (cerebrum and cerebellum) of rats. In our laboratory, studies on miRNA expression in different brain regions responsive to exercise with different intensities are currently under the way. The regulatory mechanisms of brain gene expression in response to exercise may be complex, but miRNA-mediated gene regulation can provide the important clues to understand its complex mechanisms. In this study, we identified 34 exercise-responsive miRNAs from rat brain and analyzed their functions. Although these results are still preliminary, this may lay a foundation for further elucidation of the complex regulatory mechanisms in brain responsive to exercise from the perspective of miRNA regulation. Further analysis of the function of these exercise-responsive miRNAs and their regulatory network will be our next work.

## Conclusion

A total of 2109 known and 55 novel candidate miRNAs were identified from the HIST and NC libraries of rat brain. Differential expression analysis showed that 34 HIST-responsive miRNAs were discovered, including 16 up-regulated and 18 down-regulated miRNAs. This study demonstrated that physical exercise could change the expression profiles of miRNAs in rat brain and that exercise-responsive miRNAs could regulate the expression of brain genes in response to exercise. Our findings lay a foundation for the further elucidation of the complex regulatory mechanisms responsive to exercise in the brain from the perspective of miRNA regulation.

## Additional files

**Additional file 1: Table S1.** The primers used in qRT-PCR.

**Additional file 2: Table S2.** Known miRNAs identified in HIST and NC libraries.

**Additional file 3: Table S3.** Novel candidate miRNAs identified from HIST and NC libraries.

**Additional file 4: Table S4.** Significantly differentially expressed miRNAs between HIST and NC.

**Additional file 5: Table S5.** Target genes predicted for significantly differentially expressed miRNAs.

## Abbreviations

HIST: high-intensity intermittent swimming training
NC: normal control
miRNA: microRNA
sRNA: small RNA
BDNF: brain-derived neurotrophic factor
IGF-1: insulin-like growth factor 1
NGF: nerve growth factor
NT-3: neurotrophin-3
NT-4: neurotrophin-4
5-HT: 5-hydroxytryptamine
DP: dopamine
VEGF: vascular endothelial growth factor
VGF: vascular growth factor
NMDAR1: *N*-methyl-d-aspartate receptor 1
BLAST: basic local alignment search tool
TPM: transcripts per million
MFE: minimum free energy
MFEI: minimum free energy index
RT-PCR: reverse-transcription PCR
qRT-PCR: quantitative real-time reverse-transcription PCR
SABC: streptavidin–biotin complex
DAB: diaminobenzidine
